# The Effect of MMP-2 Inhibitor 1 on Osteogenesis and Angiogenesis During Bone Regeneration

**DOI:** 10.3389/fcell.2020.596783

**Published:** 2021-01-22

**Authors:** Liangjun Jiang, Kunkun Sheng, Cong Wang, Deting Xue, Zhijun Pan

**Affiliations:** ^1^Department of Orthopedics, Second Affiliated Hospital, Zhejiang University School of Medicine, Hangzhou, China; ^2^Orthopedics Research Institute of Zhejiang University, Hangzhou, China

**Keywords:** matrix metalloproteinase 2, MMP-2 inhibitor 1, osteogenic differentiation, angiogenic, bone healing

## Abstract

Bone regeneration is a popular research focus around the world. Recent studies have suggested that the formation of a vascular network as well as intrinsic osteogenic ability is important for bone regeneration. Here, we show for the first time that matrix metalloproteinase (MMP) 2 inhibitor 1 (MMP2-I1) has a positive role in the osteogenesis of human bone marrow mesenchymal stem cells (hBMSCs) and angiogenesis of human vascular endothelial cells (HUVECs). MMP2-I1 activated the p38/mitogen-activated protein kinase signaling pathway to promote the osteogenesis of hBMSCs, and promoted the angiogenesis of HUVECs via the hypoxia-inducible factor-1α signaling pathway. We also found that MMP2-I1 enhanced bone formation using a rat tibial defect model and prevented bone loss using an ovariectomy-induced mouse model of osteoporosis. Data from the mouse model demonstrated that MMP2-I1 generated more type H vessels (CD31^hi^Emcn^hi^) when preventing bone loss. These results provide important insights into the regulatory effects of MMP2-I1 on bone regeneration.

## Introduction

Bone loss and formation are crucial issues in orthopedics (Loi et al., 2016). Bone defects or fracture nonunion are widespread and can be caused by trauma, infections, surgery, or tumor resection (Giannoudis et al., 2011). The incidence of fracture nonunion is 2.5–46% and depends on the anatomical site, fracture severity, and the injury of vascular structures and soft tissues (Zong et al., 2010). From a physiological viewpoint, osteogenic cells, growth factors, and the extracellular matrix play important roles in the process of bone repair. There has been great focus on the interactions among mesenchymal stem cells, inflammatory cells, and angiogenic cells with bone repair. The connection between osteogenesis and angiogenesis is evident during the healing of bone fractures (Dickson et al., 1995). Angiogenesis is one of the key components during bone repair, and the formation of new blood vessels in the fracture site is required for supplying nutrients, growth factors, and osteogenic cells for bone repair (Saran et al., 2014).

The matrix metalloproteinase (MMP) family of enzymes contributes to both normal and pathological tissue remodeling. As regulatory molecules, MMPs not only play the role of enzyme cascade, but also produce fragments with enhanced or reduced biological effects by processing matrix proteins, growth factors and cytokines. MMPs are members of a family of over 26 Zn-dependent endopeptidases that share a similar structure (Hernandez-Perez and Mahalingam, 2012). MMP-1 can degrade a broad range of substrates and contribute to tumor growth and tumor formation (Vincenti et al., 1996; Galateau-Salle et al., 2000; Egeblad and Werb, 2002). MMP-2 can degrade fibrillar collagen, elastin, insulin growth factor binding proteins, and the fibroblast growth factor receptor, and can activate MMP-1,−9, and−13 (Yasmin et al., 2005; Chang et al., 2013; Li and Sun, 2017; Lorenc et al., 2018). Itagaki et al. (2008) reported that increased levels of MMP-2 mRNA, which was extracted from tissue that filled the parietal bone defect, increased MMP-2 expression during the first 2 weeks, then decreased until 24 weeks. Parikka et al. (2005) found that MMP-2 and other MMPs mediated the process of collagen cleavage, an important step coupling bone formation to bone resorption. Another study reported that MMP-2 activity was higher during chondrogenic differentiation, which could be a candidate for inducing this process (Arai et al., 2016). Several studies have reported that MMP-2 is a promising target in the prevention of vascular calcifications (Aoshima et al., 2012; Liu et al., 2015; Hecht et al., 2016). These studies suggested that MMP-2 might be involved in the process of bone repair. However, studies of MMP-2 and MMP-2 inhibitors have mainly focused on cell migration and many cancers (Song et al., 2009; Hsu et al., 2013; Pan et al., 2018), and few studies of the effects of MMP-2 or MMP-2 inhibitors on bone healing have been reported. MMP-2-I1 is a long-chain unsaturated fatty acid that preferentially targets the two gelatinases, MMP-2 and MMP-9, which is orally active in animal models (Tamura et al., 1998; Berton et al., 2001).

In the present study, we investigated the effects and mechanisms of MMP-2 inhibitor 1 (MMP2-I1) on bone repair. The results showed that MMP2-I1 promoted the osteogenesis of human bone marrow mesenchymal stem cells (hBMSCs) through activation of the p38/mitogen-activated protein kinase (MAPK) signaling pathway, and enhanced angiogenesis of human vascular endothelial cells (HUVECs) via the hypoxia-inducible factor (HIF)-1α signaling pathway, which ultimately accelerated bone formation. Furthermore, the effects of MMP2-I1 on the rat tibial defect model and ovariectomy (OVX)-induced mouse model of osteoporosis confirmed its positive role in bone repair.

## Materials and Methods

### Cell Culture and Reagents

hBMSCs were isolated from bone marrow as previously described (Chen et al., 2018). Cell culture and passage are consistent with the above reference.

HUVECs were obtained from Lonza (Basel, Switzerland) and cultured in RPMI1640 medium with 10% fetal bovine serum, 1% penicillin-streptomycin, 0.1 mg/mL heparin, and 0.05 mg/mL endothelial cell growth supplement.

MMP2-I1 was purchased from Merck KGaA (Darmstadt, Germany). p38 inhibitor (SB203580; Selleck, Shanghai, China) and HIF-1α inhibitor (KC7F2; Selleck) were used in this study.

### Osteogenic Differentiation and Cell Treatment

Osteogenesis of hBMSCs was induced by osteogenic differentiation medium (ODM) (Cyagen Biosciences, Guangzhou, China), which consisted of hBMSC osteogenic differentiation basal medium,10% fetal bovine serum, 1% penicillin–streptomycin, 100 nM dexamethasone, 10 mM b-glycerophosphate, and 0.05 mM L-ascorbic acid-2-phosphate. hBMSCs were cultured on 6-well plate (Corning, NY, USA). After cells reached confluence, the culture medium was aspirated, and ODM was added. The ODM was changed every 3 days. After 3 or 7 days of osteogenic differentiated culture, the cells were collected and tested.

### Cell Viability Assay

To assess the proliferation effects of MMP2-I1, cells were seeded into 96-well plates (5,000 cells/well) and cultured for 24 h. After 24 h, MMP2-I1 were added to wells and incubated for the indicated times. Cell viability was measured using the Cell Counting Kit-8 (CCK-8) assay (Dojindo Laboratories, Shanghai, China) according to the manufacturer's instructions.

The EdU-555 fluorescence staining kit (Beyotime, Shanghai, China) was used to assess the proliferation effects of MMP2-I1. Cells were seeded into plates and cultured for 24 h. After 24 h, MMP2-I1 were added to wells and incubated for the indicated times. Prior to measurements, 10 μM EdU solution was added to each well according to the manufacturer's instructions. Samples were observed under a fluorescence microscope (Leica, Wetzlar, Germany).

### Alkaline Phosphatase (ALP) Staining and ALP Activity Assay

hBMSCs were cultured with ODM and MMP2-I1 at different concentrations for 3 days. For ALP staining, we processed the cells according to the manufacturer's instructions using the BCIP/NBT Alkaline Phosphatase Color Development Kit (Beyotime). For measurement of ALP activity, we processed the cells according to the manufacturer's instructions using the ALP Activity Assay (Beyotime).

### Alizarin Red Staining (ARS) and Quantification

After osteogenic differentiation, mineral deposition was assessed by ARS (Cyagen Biosciences). Briefly, Cells were fixed in 4% paraformaldehyde for 15 min then incubated with Alizarin Red for 20 min at room temperature. The quantification of ARS was described previously (Chen et al., 2018).

### RNA Isolation and Quantitative Reverse Transcription Polymerase Chain Reaction (qPCR)

Total cellular RNA was isolated using RNAiso reagent (TaKaRa Bio, Tokyo, Japan). The Prime Script RT Master Mix (TaKaRa Bio) and Double-Strand cDNA Synthesis Kit (TaKaRa Bio) was used according to the manufacturer's instructions. The details were described previously (Chen et al., 2018). The primer sequences used are shown in Table 1.

**Table 1:** Sequences of primers for quantitative real-time polymerase chain reaction.

**Gene name**	**Forward primer sequence (5^**′**^ to 3^**′**^)**	**Reverse primer sequence (5^**′**^ to 3^**′**^)**
RUNX2	ACTTCCTGTGCTCGGTGCT	GACGGTTATGGTCAAGGTGAA
OSX	CCTGCGACTGCCCTAATT	GCGAAGCCTTGCCATACA
COL1A1	GAGAGCATGACCGATGGATT	CCTTCTTGAGGTTGCCAGTC
VEGF	GCTGTCTTGGGTGCATTGG	GCAGCCTGGGACCACTTG
CD31	AACAGTGTTGACATGAAGAGCC	TGTAAAACAGCACGTCATCCTT
Emcn	AGCAACCAGCCGGTCTTATTC	AGCACATTCGGTACAAACCCA
GAPDH	GAAAGCCTGCCGGTGACTAA	TGGAATTTGCCATGGGTGGA

### Western Blot Analysis

Cells were lysed in radioimmunoprecipitation buffer supplemented with proteasome inhibitor and phosphatase inhibitor cocktail (Beyotime). The detail protocol was described previously (Chen et al., 2018). The antibodies used are listed in Table 2.

**Table 2:** The antibodies used in this study.

**Antibody**	**Dilution Rate**	**Company and catalog number**
RUNX2	1:1,000	CST/12556
COL1A1	1:2,000	Abcam/ab34710
OSX	1:1,000	Affinity/DF7731
VEGF	1:1,000	Affinity/AF5131
CD31	1:1,000	Affinity/AF6191
Emcn	1:50	Abcam/ab106100
p-ERK	1:1,000	CST/4370
t-ERK	1:1,000	CST/4695
p-P38	1:1,000	CST/9215
t-P38	1:1,000	CST/9212
p-JNK	1:1,000	Abcam/ab124956
t-JNK	1:2,000	CST/9252
a-β-catenin	1:1,000	CST/19807
t-β-catenin	1:1,000	CST/8480
p-P65	1:1,000	CST/3033
t-P65	1:1,000	CST/8242
p-SMAD	1:1,000	CST/13820
HIF-1α	1:1,000	CST/36169
GAPDH	1:1,500	CST/5174

### Migration Assay

For the scratch wound assay, HUVECs were seeded into plates and grown until confluent. The cells were wounded with a pipette tip, and the cells were incubated in RPMI1640 medium with different concentrations of MMP2-I1. Images of the wounds were acquired immediately and after 12 h. We used ImageJ software (National Institutes of Health, Bethesda, MD, USA) to calculate the rate of migration area as follows: migration area (%) = (A0 - An)/A0 × 100, where A0 represented the area of the initial wound area, and An represented the residual area of the wound at the metering point.

For the Transwell migration assay, HUVECs were suspended at a density of 5 × 10^4^/200 μL of RPMI1640 media and loaded into the upper chamber of a 24-well Transwell plate with 8 μm pore polyester membrane inserted (Corning). Then, the RPMI1640 medium with MMP2-I1 was loaded into the lower chamber. After 6 h, the migrated cells that passed through the membrane pores were stained with crystal violet for 5 min and counted using an optical microscope (Leica).

### Tube Formation Assay

HUVECs (6.5 × 10^4^ cells/well) were seeded onto Matrigel-coated 48-well plates and cultured in RPMI1640 medium with MMP2-I1 for 12 h. Cells were observed with an inverted microscope (Leica). All parameters (meshes and master junction) revealing the ability of HUVECs to form tubes were measured using ImageJ software.

### *In vitro* Fibrin Gel Angiogenesis Assay

For fibrin gel angiogenesis assays, HUVECs were seeded onto Cytodex 3 micro-carrier beads (Sigma-Aldrich) at a concentration of ~400 cells per bead for 4 h and allowed to adhere overnight. after overnight culture, the beads were suspended in a 2 mg/mL fibrinogen solution (Sangon Biotech, Shanghai, China) containing 0.15 units/mL of aprotinin (Sangon Biotech) and 0.625 units/mL of thrombin (Sangon Biotech) and allowed to clot in 96-well plates. After the fibrinogen solidified, 150 μL of RPMI1640 medium under different concentrations of MMP2-I1 was added. HUVECs sprouting was monitored for 7 days and scored for total branching length using ImageJ software.

### Small Interference RNA (siRNA) Transfection

MMP-2 siRNA was synthesized by Sangon Biotech. The negative control (NC) comprising of a 21-bp non-targeting sequence functions to distinguish sequence-specific silencing from non-specific effects. MMP-2 and NC siRNA sequences were as follows: MMP-2, sense strand: 5′- UGGAUUUGUACCAUUCUUCUG-3′, antisense strand: 5′-GAAGAAUGGUACAAAUCCAAG-3′; NC, sense strand: 5′-UUCUCCGAACGUGUCACGUTT-3′, antisense strand: 5′-ACGUG ACACGUUCGGAGAATT-3′. siRNA was added to the Opti-MEM (ThermoFisher, Waltham, MA, USA) containing Lipofectamine 2000 (ThermoFisher) to transfect cells. The medium was changed 6 h after cell transfection for further culture. The knockdown efficiency of mRNA level was detected by qPCR after 48 h, and the knockdown efficiency of protein level was detected by western blot after 72 h.

### MMP-2 and MMP-9 Activity Assay

To assess the inhibitory effects of MMP2-I1 on MMP-2 and MMP9 activity, cells were seeded into 96-well plates (5,000 cells/well) and cultured for 24 h. After 24 h, hBMSCs were cultured with ODM and MMP2-I1 at different concentrations for 3 days. MMP-2 and MMP9 activity were measured using the MMP-2 Biotrak Activity Assay System (GE Life, Shanghai, China) and MMP9 Inhibitor Screening Assay Kit (Abcam, Shanghai, China) according to the manufacturer's instructions.

### *In vivo* Evaluation

All Sprague–Dawley rats and C57BL/J6 mice were supplied by the Academy of Medical Sciences of Zhejiang Province. We performed animal experiments accordance with the Animal Care and Use Committee guidelines of Zhejiang Province. All experimental procedures were approved by the Institutional Animal Care and Use Committee at Zhejiang University.

### Rat Tibial Defect Model

Tibial defects were generated in 8-week-old male Sprague–Dawley rats. The details were described previously (Chen et al., 2017). The 18 defects in 18 rats were randomized into three groups. In the blank group (*n* = 6), nothing was grafted onto the defect site; in the gel group (*n* = 6), the defect areas were filled with hydrogel (Beaver, Suzhou, China) only; and in the MMP2-I1 group (*n* = 6), the hydrogel with MMP2-I1 was implanted into the defects. The hydrogel was fabricated from Type I collagen, chondroitin sulfate and a degradable copolymer, which is injectable, rapid gelling and capable of releasing MMP2-I1.

### OVX-Induced Osteoporosis Model

To assess the effects of MMP2-I1 on osteoporosis *in vivo*, we established an OVX-induced osteoporosis model. Briefly, 30 healthy 8-week-old female C57BL/J6 mice were randomly assigned to three groups: control with PBS (sham), OVX with PBS (vehicle), and OVX with MMP2-I1 (MMP2-I1). Mice in the MMP2-I1 group were injected intraperitoneally with 2 mg/kg MMP2-I1, twice per week for 6 weeks. Mice in the sham and vehicle groups were injected with PBS. Vertebral bodies and femurs were excised and fixed in 4% paraformaldehyde for microcomputed tomography (CT) and histological evaluation.

### CT Evaluation

Samples (rats tibia and mice femurs/vertebral bodies) were collected 6 weeks after surgery. All samples were scanned using a CT 100 imaging system as described previously (Chen et al., 2017). The bone volume fraction (BV/TV), trabecular number (Tb.N), trabecular thickness (Tb.Th), and trabecular separation (Tb.Sp) were calculated by three-dimensional standard microstructural analyses.

### Histological Evaluation

The samples (rats tibia and mice femur) were fixed in 4% paraformaldehyde for 48 h at room temperature and decalcified. After embedding in paraffin, serial sections were cut and mounted on polylysine-coated slides. Hematoxylin and eosin (H&E) and Masson staining were performed separately on consecutive tissue sections.

Tissue sections (mice femur) were permeabilised and blocked. Fixed tissues were incubated overnight with anti-CD31 (1:3,200; Cell Signaling Technology, Danvers, MA, USA) and anti-EMCN (1:200; Abcam). Tissues were incubated with secondary antibody, and the nuclei were stained with 4′,6-diamidino-2-phenylindole.

### Statistical Analysis

Statistical analysis was performed using SPSS statistical software for Windows, version 17.0 (IBM, Armonk, NY, USA). All experiments were performed at least in triplicate, and the data are presented as the means ± standard deviation.

Statistical significance was determined using a two-tailed Student's *t*-test when two groups were compared, one-way analysis of variance (ANOVA) followed by Bonferroni's *post-hoc* test when more than two groups were compared, and two-way ANOVA followed by Bonferroni's multiple comparisons *post-hoc* test when treatment groups at different time points were compared. A value of *P* ≤ 0.05 was considered to represent a statistically significant difference.

## Results

### MMP2-I1 Did Not Affect hBMSCs Proliferation

**Figure 1:**
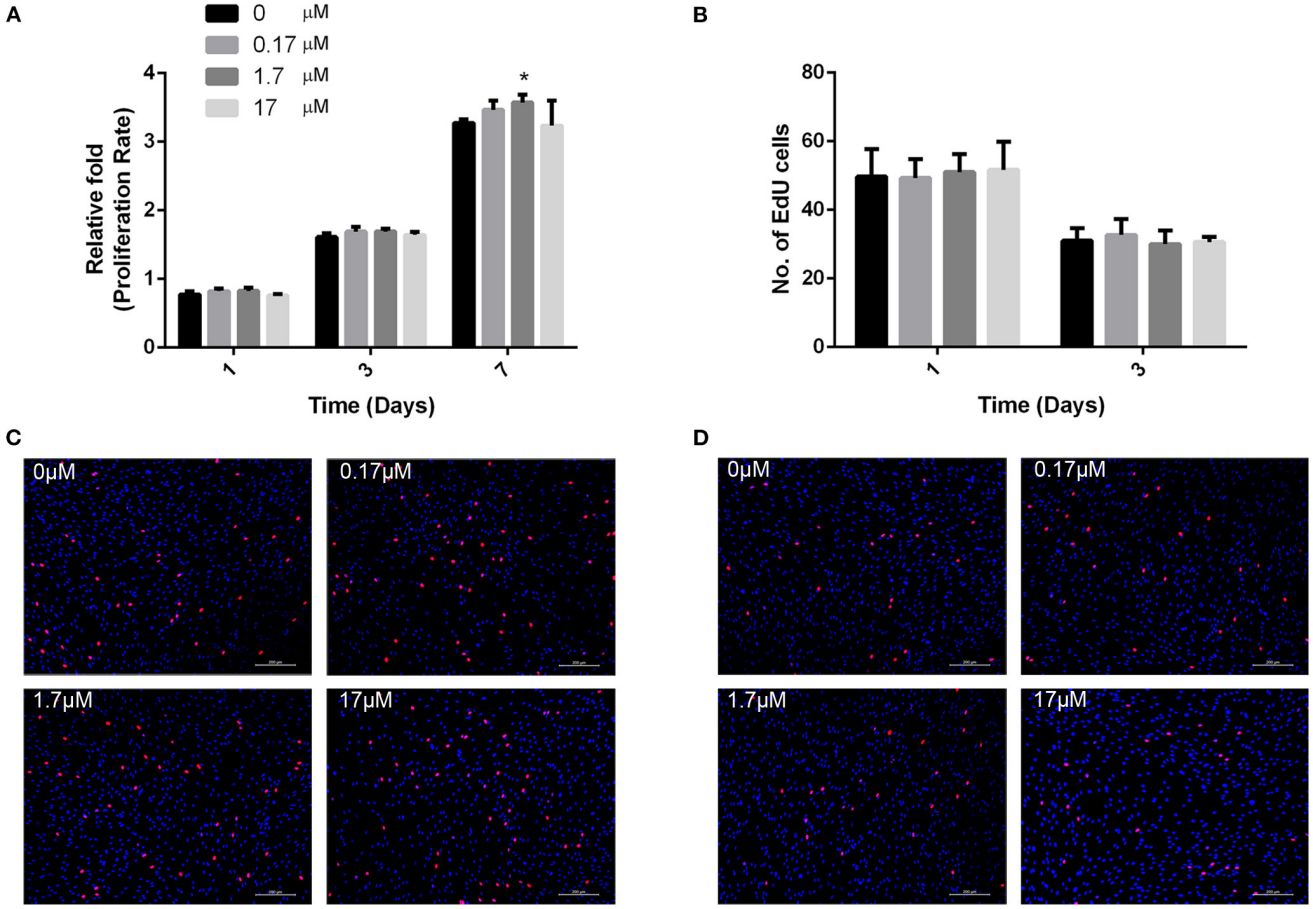
MMP2-I1 did not affect hBMSCs proliferation. **(A)** The hBMSCs were incubated with different concentrations of MMP2-I1 for 1, 3, and 7 days, then CCK-8 assay was performed to test the proliferation. **(B)** Quantification of EdU-555 positive cells in different groups at days 1 and 3. **(C,D)** EdU-555 fluorescence staining of hBMSCs in different groups at days 1 and 3. All the data were confirmed by three repeated tests. Data were mean ± S.D. *p < 0.05 vs. the control group. Scale bar = 200 μm.

To determine whether MMP2-I1 influenced the proliferation of hBMSCs, we used a CCK-8 assay and EdU-555 fluorescence staining. The effects of MMP2-I1 on proliferation of hBMSCs are shown in Figure 1A. The proliferation of hBMSCs was not affected following treatment with MMP2-I1 at concentrations between 0.17 and 17 μM at days 1, 3, and 7. Although there was an upward trend on day 7, we considered it to be very slight. Moreover, no significant difference was detected with EdU-555 fluorescence staining of cells in different groups at days 1 and 3 (Figures 1B–D), which was consistent with the results of the CCK-8 assay.

### The Effects of MMP2-I1 on hBMSCs ALP and Calcium Deposits

**Figure 2:**
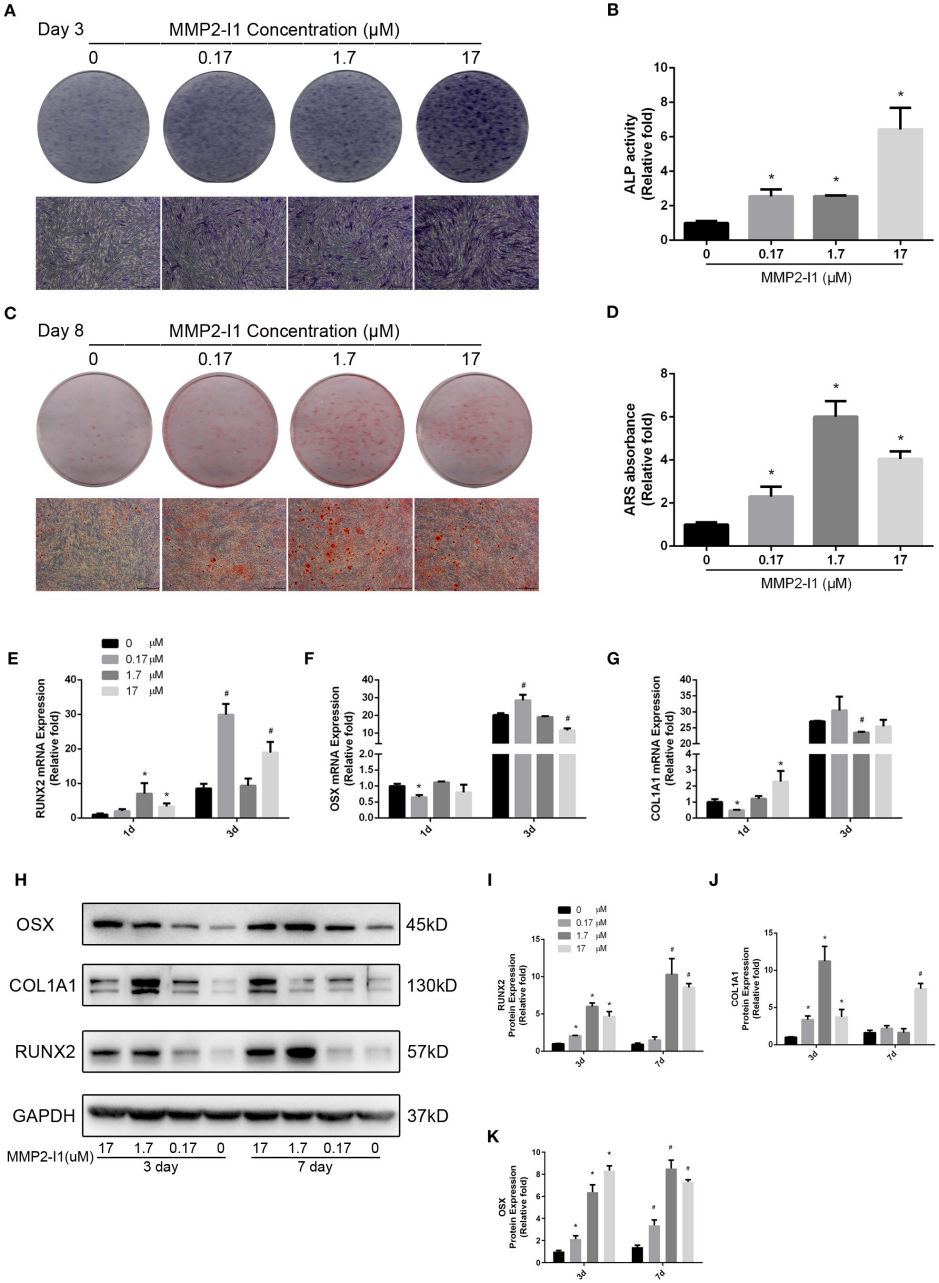
The effects of MMP2-I1 on osteogenesis of hBMSCs. **(A)** ALP in hBMSCs was stained after the osteogenic differentiation for 3 days. **(B)** The ALP activity of hBMSCs after the osteogenic differentiation for 3 days. **(C)** Alizarin red staining in hBMSCs after the osteogenic differentiation for 8 days. **(D)** Mineralization was quantified by the extraction of ARS stain cells. **(E)** The expression of *RUNX2* mRNA was determined by qPCR at days 1 and 3 of osteogenic differentiation. **(F)** The expression of *OSX* mRNA. **(G)** The expression of *COL1A1* mRNA. **(H–K)** The expression of RUNX2, COL1A1 and OSX proteins were determined by Western blot analysis after osteogenic differentiation for 3 and 7 days. All the data were confirmed by three repeated tests. Data were mean ± S.D. *p < 0.05 vs. the control group at the same day. #*p* < 0.05 vs. the control group at the same day. Scale bar = 500 μm.

ALP activity of hBMSCs was detected during osteogenic differentiation with MMP2-I1 at 3 days. Higher ALP activity was observed in the 0.17–17 μM MMP2-I1 groups (Figure 2B), especially in the 17 μM MMP2-I1 group. ALP staining produced similar results (Figure 2A). More calcium deposits of hBMSCs were observed in the 0.17–17 μM treatment groups than in the control group at 8 days (Figure 2C). The most obvious deposit is in 1.7 μM group. The quantitative analysis produced similar results (Figure 2D).

### The Effects of MMP2-I1 on the Osteo-Specific Genes and Proteins

To assess the effect of MMP2-I1 in osteogenesis of hBMSCs, the osteo-specific genes and proteins, including *RUNX2, OSX*, and collagen type I alpha 1 chain (*COL1A1*) were determined by qPCR and western blot analysis. MMP2-I1 increased the expression of *RUNX2* (1 and 3 days), *OSX* (3 days), and *COL1A1* (1 days) (Figures 2E–G). The most obvious increase was found in the RUNX2 mRNA at 1 and 3 days of osteogenic differentiation.

Western blotting analysis revealed that expression of the RUNX2 protein was increased by treatment of MMP2-I1 at 3 and 7 days, especially in the range of 1.7–17 μM. COL1A1 and OSX protein expression was also significantly increased by MMP2-I1 at 3 and 7 days (Figures 2H–K). We found some spike in the expression of RUNX2 at 7 days under the MMP2-I1 concentration of 1.7 μM and in the expression of COL1A1 at 3 days under the MMP2-I1 concentration of 1.7 μM and 7 days under that of 17 μM. MMP2-I1 promote OSX protein expression in a dose-dependent manner at 3 days. Together, these data showed that MMP2-I1 enhanced the osteogenic differentiation of hBMSCs.

We silenced MMP-2 with MMP-2 siRNA in the hBMSCs. Remarkably, the MMP-2 silenced hBMSCs showed the higher ALP activity and more calcium deposits than the control cells (Supplementary Figures 1A–D). In addition, the MMP-2 silenced hBMSCs increased the RUNX2, COL1A1, and OSX protein expression during osteogenic differentiation (Supplementary Figures 1E–H). These data were consistent with the above results.

### The Effects of MMP2-I1 on the Angiogenesis of HUVECs

**Figure 3:**
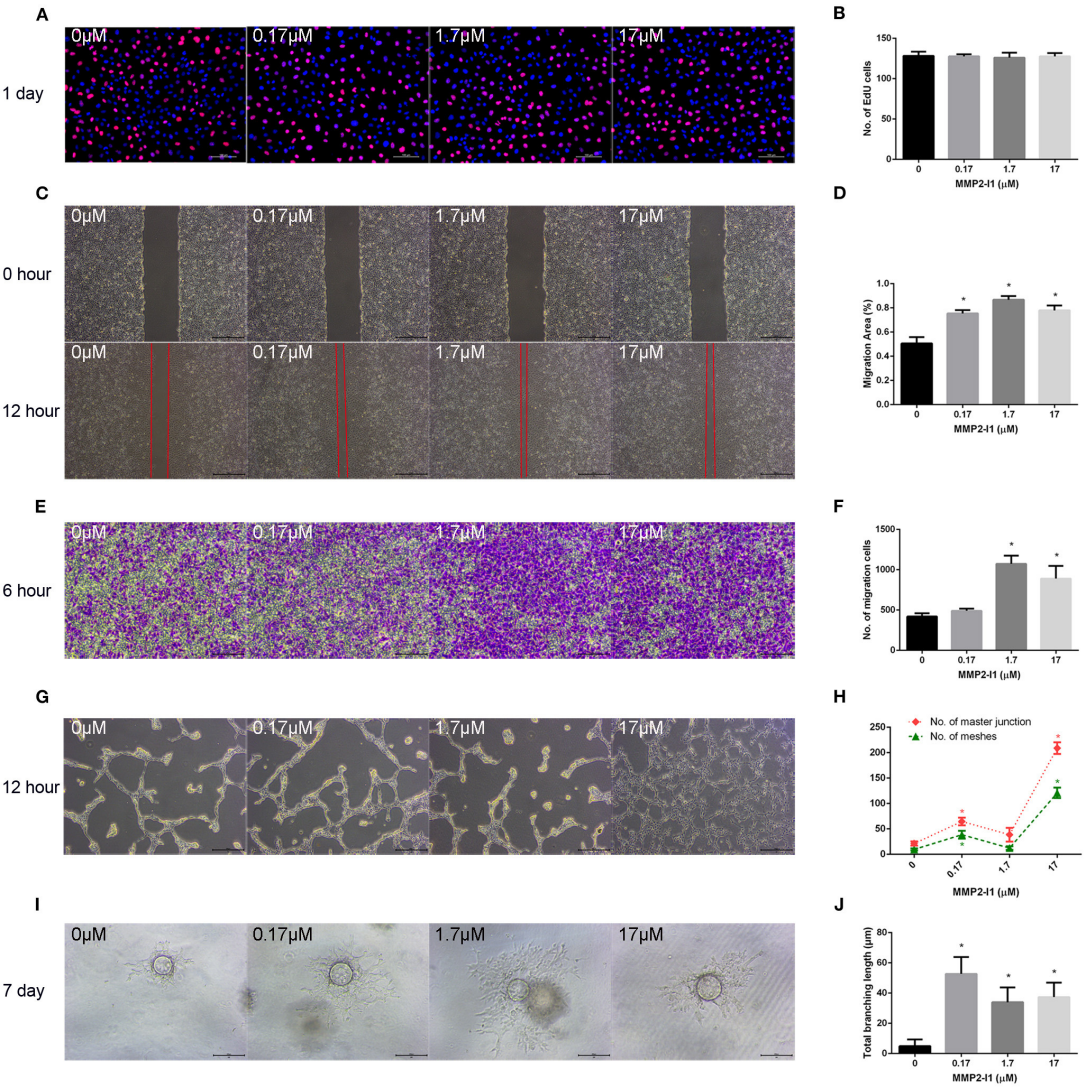
The effects of MMP2-I1 on angiogenesis of HUVECs. HUVECs were cultured in RPMI1640 medium with MMP2-I1. **(A)** EdU-555 fluorescence staining of HUVECs in different groups at day 1. Scale bar = 100 μm. **(B)** Quantification of EdU-555 positive cells. **(C)** The scratch wound assay of HUVECs at 0 and 12 h. Scale bar = 500 μm. **(D)** Quantification of the rate of migration area. **(E)** The transwell® migration assay of HUVECs at 6 h. Scale bar = 200 μm. **(F)** Quantification of the migrated cells. **(G)** The tube formation assay of HUVECs at 12 h. Scale bar = 500 μm. **(H)** Quantification of meshes and master junction. **(I)** The fibrin gel angiogenesis assay of HUVECs at 7 d. Scale bar = 200 μm. **(J)** Quantification of total branching length. All the data were confirmed by three repeated tests. Data were mean ± S.D. *p < 0.05 vs. the control group.

To evaluate the role of MMP2-I1 in angiogenesis, HUVECs were incubated with different concentrations of MMP2-I1 for a series of angiogenesis-related functional assays. Figures 3A,B show that MMP2-I1 did not influence the proliferation of HUVECs, which were stained with EdU-555. The scratch wound and Transwell assays showed that MMP2-I1 treatment increased the migration of HUVECs compared to the control group, especially at 1.7–17 μM (Figures 3C–F). For the scratch wound assay, the area not covered by cells in the scratch area at 1.7 and 17 μM was significantly smaller than that in the control group. For the Transwell migration assay, more HUVECs were passed through the membrane pores at 1.7 and 17 μM than that in the control group. HUVECs treated with MMP2-I1 formed a higher number of capillary tube-like structures (meshes and master junctions) on Matrigel compared with the control group, especially at 17 μM (Figures 3G,H). Using a spheroid sprouting assay, we found that MMP2-I1 stimulated HUVECs sprouting in a fibrin gel, especially at 0.17 μM (Figures 3I,J).

### The Effects of MMP2-I1 on the Angio-Specific Genes and Proteins

**Figure 4:**
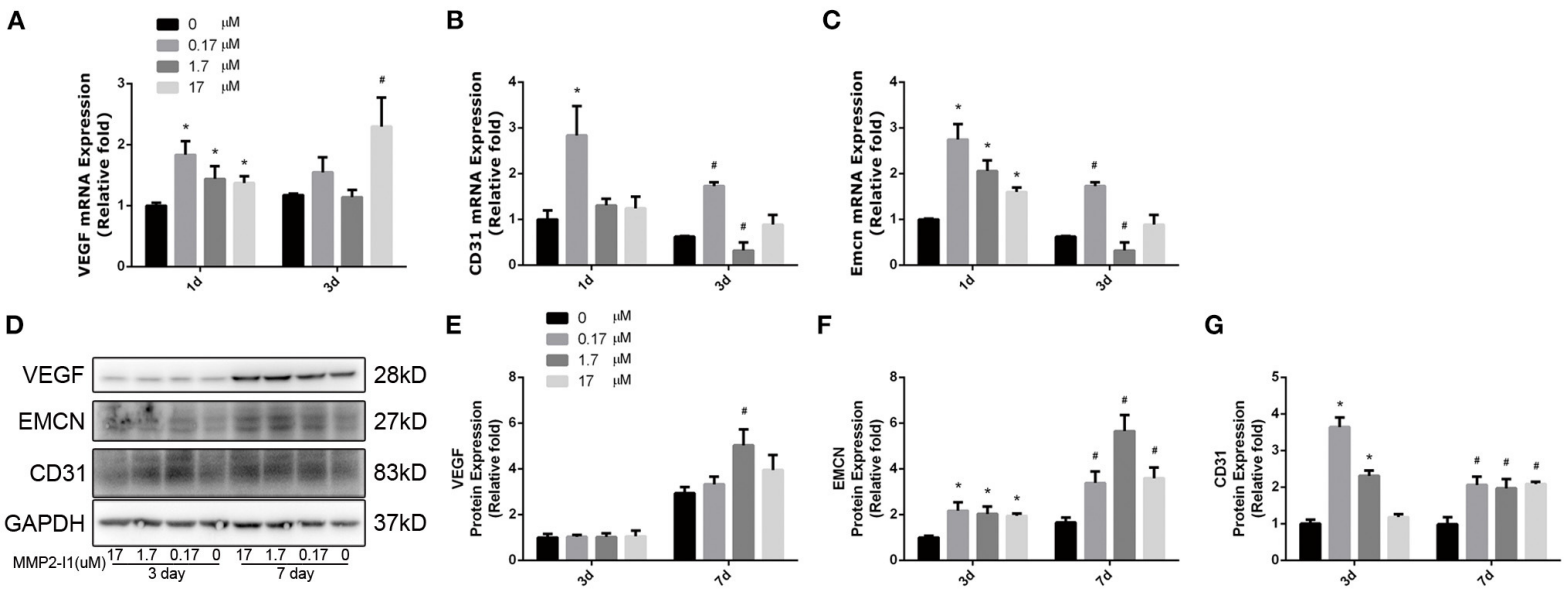
The effects of MMP2-I1 on levels of angio-specific genes and proteins of HUVECs. **(A)** The expression of *VEGF* mRNA was determined by qPCR at days 1 and 3. **(B)** The expression of *CD31* mRNA. **(C)** The expression of *EMCN* mRNA. **(D–G)** The expression of VEGF, EMCN and CD31 proteins were determined by Western blot analysis for 3 and 7 days. All the data were confirmed by three repeated tests. Data were mean ± S.D. *p < 0.05 vs. the control group at the same day. #*p* < 0.05 vs. the control group at the same day.

To assess the effect of MMP2-I1 in angiogenesis of HUVECs, the angio-specific genes and proteins, including vascular endothelial growth factor (*VEGF*), *CD31* (platelet and endothelial cell adhesion molecule 1, PECAM1), and endomucin (*EMCN*) were determined by qPCR and western blot analysis. HUVECs were cultured in RPMI1640 medium with MMP2-I1 for 1 and 3 days. The media was changed every 3 days. MMP2-I1 increased the expression of *VEGF* (1 and 3 days), *CD31* (1 and 3 days), and *EMCN* (1 and 3 days) (Figures 4A–C), especially at 0.17 μM.

HUVECs were cultured in RPMI1640 medium with MMP2-I1 for 3 and 7 days. The media was changed every 3 days. Notably, western blot analysis revealed that VEGF protein expression was not increased by MMP2-I1 at 3 and 7 days, except at 1.7 μM at 7 days. Expression of CD31 and EMCN was significantly increased in the MMP2-I1 treatment groups at 3 and 7 days compared to the control group (Figures 4D–G). The MMP-2 silenced HUVECs with siRNA showed the similar results (Supplementary Figure 2). Taken together, these data showed that MMP2-I1 promoted the angiogenesis of HUVECs.

### Activation of p38/MAPK Signaling in hBMSCs and HIF-1α Signaling in HUVECs

**Figure 5:**
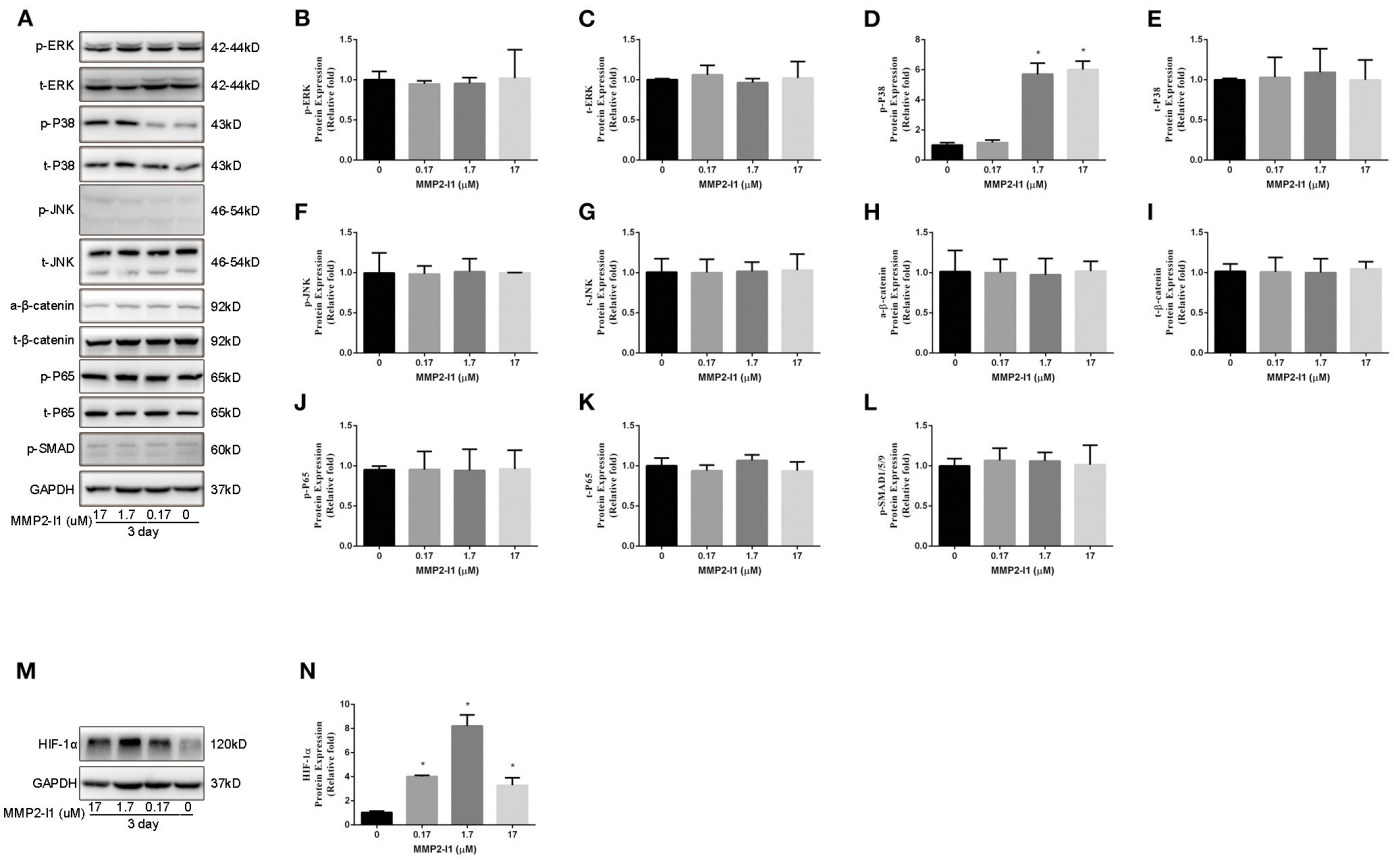
Activation of p38/MAPK signaling in hBMSCs and HIF-1α signaling in HUVECs. **(A–L)** The expression of MAPK, Wnt/β-catenin, NF-κB, and SMAD signaling pathways proteins in hBMSCs were determined by Western blot analysis at day 3 of osteogenic differentiation. **(M,N)** The expression of HIF-1α protein in HUVECs was determined by Western blot analysis at day 3. All the data were confirmed by three repeated tests. Data were mean ± S.D. *p < 0.05 vs. the control group.

To evaluate whether MMP2-I1 influenced osteogenesis of hBMSCs through the Wnt/β-catenin, MAPK, or NF-κB signaling pathways, we inquired the effects of the MMP2-I1 using western blotting at 3 days (Figure 5A) and quantified the results (Figures 5B–L). MMP2-I1 at 1.7–17 μM induced a rapid activation of p38 (Figure 5A), leading to the phosphorylation of p38, but did not affect extracellular signal-regulated kinase (ERK), c-Jun N-terminal kinase, β-catenin, p65, or p-SMAD. These data suggested that MMP2-I1 affected the osteogenesis of hBMSCs through p38/MAPK signaling. Subsequently, we examined the angio-associated pathway protein in HUVECs, HIF-1α, and found that it was significantly upregulated by MMP2-I1 at 3 days (Figures 5M,N). Overall, these data suggested that MMP2-I1 activated HIF-1α signaling to promote angiogenesis of HUVECs.

### Inhibition of p38/MAPK Impairs Osteogenic Differentiation Induced by MMP2-I1 Treatment

**Figure 6:**
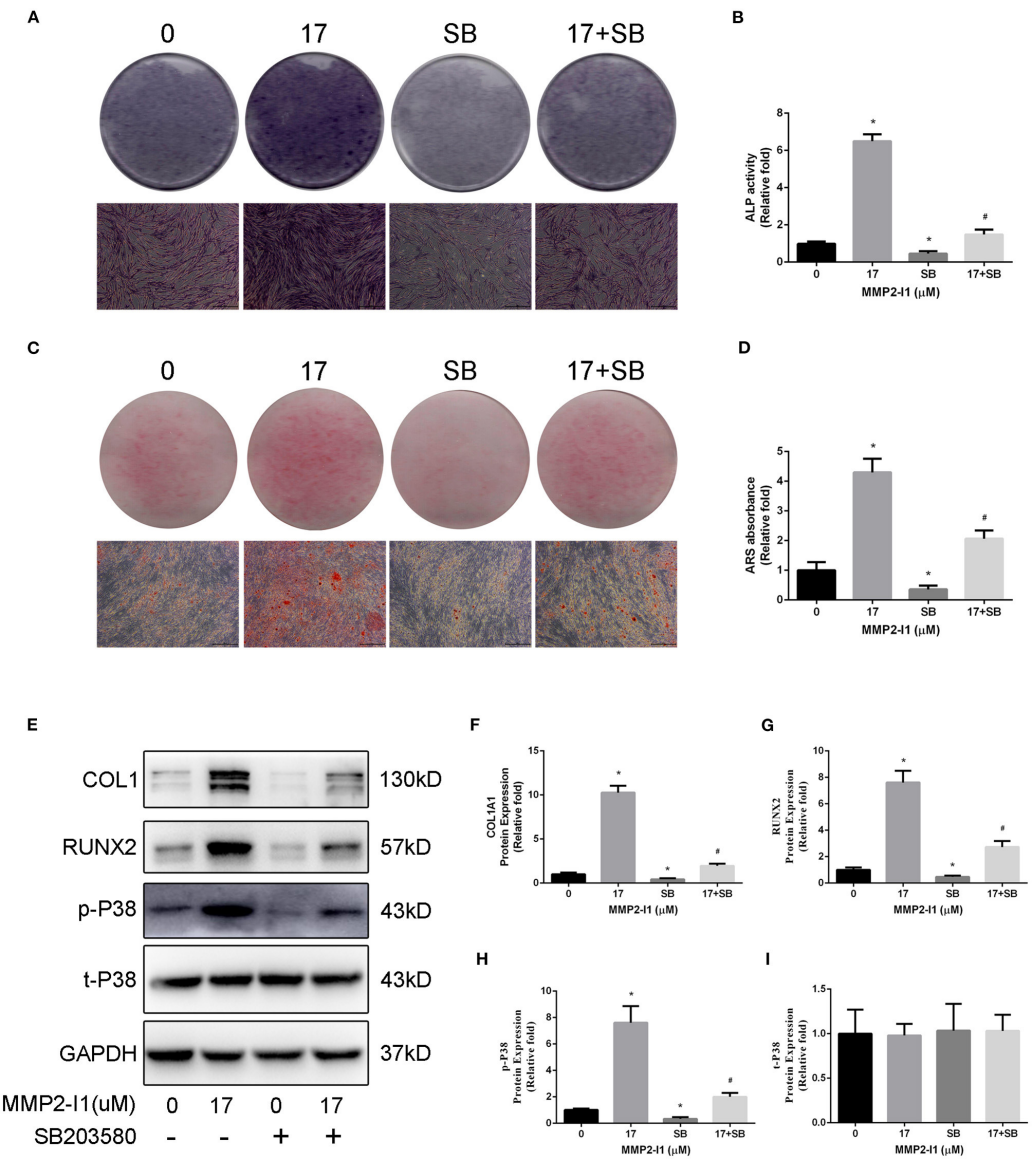
Inhibition of p38/MAPK impairs osteogenic differentiation of hBMSCs induced by MMP2-I1 treatment. To investigate the role of p38/MAPK in osteogenesis of hBMSCs, we inhibited the p38/MAPK pathway using a p38 inhibitor (SB203580). **(A,B)** ALP staining and activity at days 3 of osteogenesis. **(C,D)** Alizarin red staining and quantitation at day 8 of osteogenesis. **(E–I)** The expression of RUNX2, COL1A1, and p38/MAPK signaling pathways proteins were determined by Western blot analysis at days 3 of osteogenesis. All the data were confirmed by three repeated tests. Data were mean ± S.D. *p < 0.05 vs. the 0 group. #*p* < 0.05 vs. the SB group. SB is short for SB203580. Scale bar = 500 μm.

To further investigate the role of p38/MAPK in osteogenesis of hBMSCs, we inhibited the p38/MAPK pathway using a p38 inhibitor (SB203580). As shown in Figures 6A–D, blocking p38 decreased the levels of ALP and mineralization. After adding SB203580 in the osteogenic media, the ALP activity was decreased and the ALP staining show the same result. Moreover, SB203580 impairs the calcium deposits of MMP2-I1 promotion. Western blotting was performed to determine the extent of reduction of the phosphorylation of p38 and osteo-specific proteins at 3 days (Figures 6E–I). Blocking p38 reduced the expression of RUNX2 and COL1A1. These results confirmed that p38 played a positive role in the osteogenesis of MMP2-I1 on hBMSCs.

### Inhibition of HIF-1α Impairs Angiogenic Differentiation Induced by MMP2-I1 Treatment

**Figure 7:**
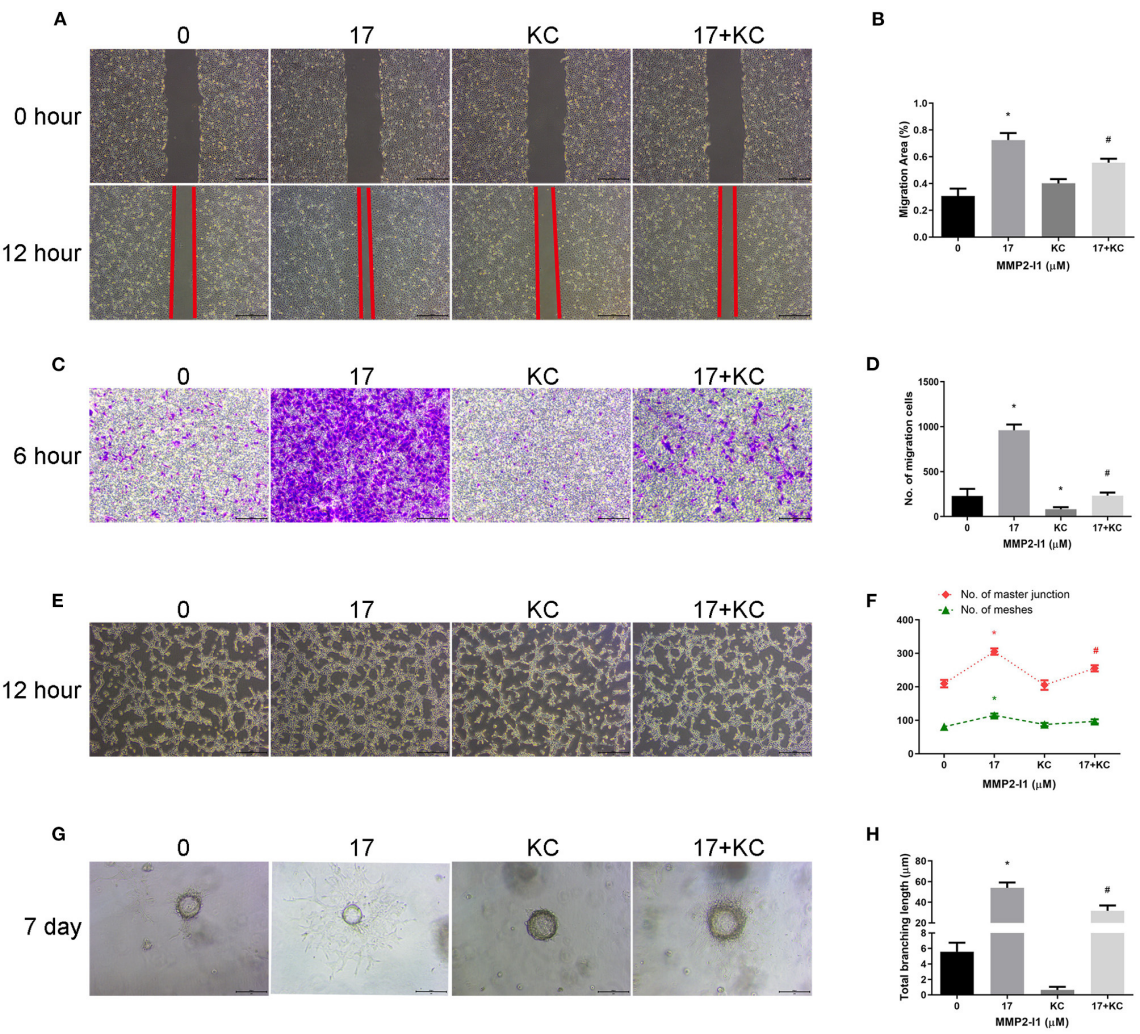
Inhibition of HIF-1α impairs angiogenic differentiation induced by MMP2-I1 treatment. To investigate the role of HIF-1α in angiogenesis of HUVECs, we inhibited the HIF-1α pathway using a specific inhibitor (KC7F2). **(A)** The scratch wound assay of HUVECs at 0 and 12 h. Scale bar = 500 μm. **(B)** Quantification of the rate of migration area. **(C)** The transwell® migration assay of HUVECs at 6 h. Scale bar = 200 μm. **(D)** Quantification of the migrated cells. **(E)** The tube formation assay of HUVECs at 12 h. Scale bar = 500 μm. **(F)** Quantification of meshes and master junction. **(G)** The fibrin gel angiogenesis assay of HUVECs at 7 d. Scale bar = 200 μm. **(H)** Quantification of total branching length. All the data were confirmed by three repeated tests. Data were mean ± S.D. *p < 0.05 vs. the 0 group. #*p* < 0.05 vs. the KC group. KC is short for KC7F2.

To further investigate the role of HIF-1α in angiogenesis of HUVECs, we inhibited the HIF-1α pathway using a HIF-1α inhibitor (KC7F2). As shown in Figures 7A–D, blocking HIF-1α decreased migration of HUVECs compared to the control group. After adding KC7F2 in the media, the tuber formation ability was mildly decreased (Figures 7E,F). Moreover, KC7F2 impairs the sprouting of MMP2-I1 promotion in a fibrin gel (Figures 7G,H). These results confirmed that HIF-1α played a positive role in the angiogenesis of MMP2-I1 on HUVECs.

### MMP2-I1 Accelerates Bone Formation in a Rat Tibial Defect Model

**Figure 8:**
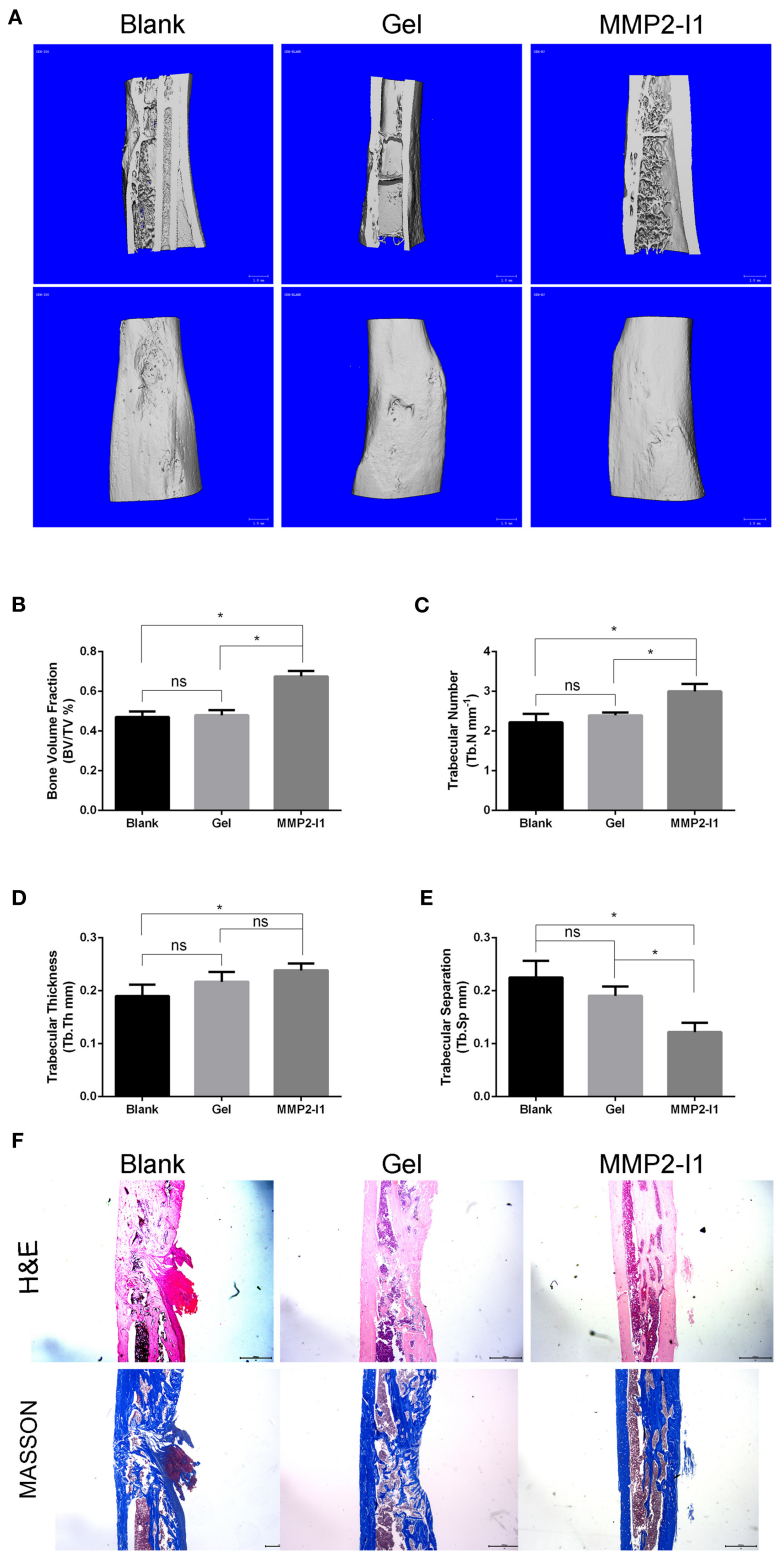
MMP2-I1 accelerates bone formation in a rat tibial defect model. Rats tibia were collected 6 weeks after surgery for CT evaluation and were decalcified for HE staining and masson staining. **(A)** Micro-CT images of tibia. **(B–E)** Micro-CT analyses of the bone volume fraction (BV/TV), trabecular number (Tb.N), trabecular thickness (Tb.Th), and trabecular separation (Tb.Sp). **(F)** HE staining and Masson staining of tibia. All the data were confirmed by three repeated tests. Data were mean ± S.D. *p < 0.05 vs. the aimed group. Blank for nothing was grafted onto the defect site; Gel for the defect areas were filled with hydrogel only; MMP2-I1 for the hydrogel with MMP2-I1 was implanted into the defects. Scale bar = 1 mm.

According to the CT taken at 6 weeks, more new bone formation was observed in the MMP2-I1 treatment group than in the other groups (Figure 8A). Quantitatively, the defects in the MMP2-I1 treatment group displayed an increase in BV/TV, Tb.N, Tb.Th, and a decrease in Tb.Sp compared with the blank group (Figures 8B–E). Sections were stained with H&E and Masson in Figure 8F. Few bridging bone formations in the defect area were observed in the blank and gel groups. In the MMP2-I1 treatment group, the defect area was almost sealed.

### MMP2-I1 Attenuates OVX-Induced Osteoporosis

**Figure 9:**
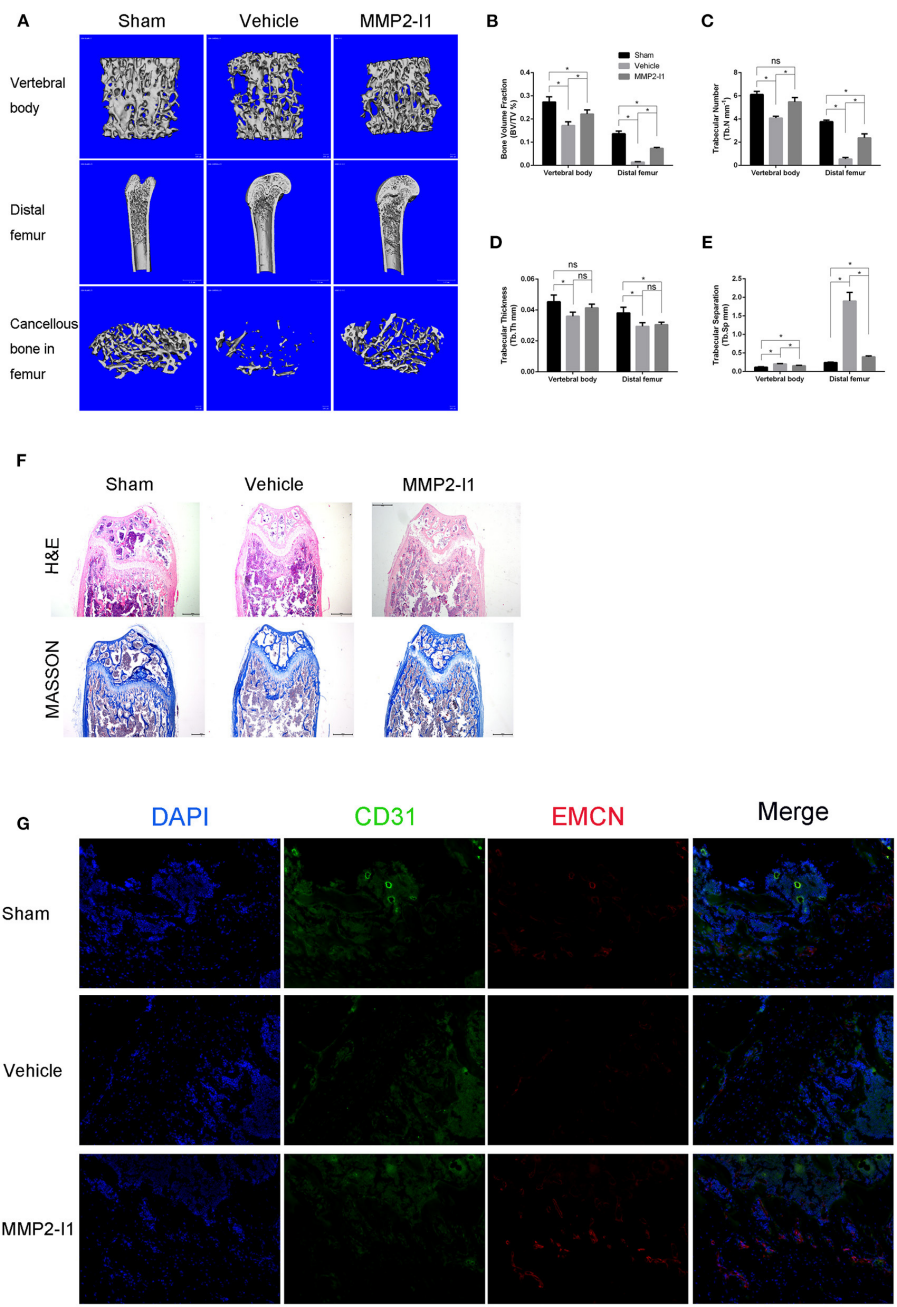
MMP2-I1 attenuates OVX-induced osteoporosis. Mice femurs and vertebral bodies were collected 6 weeks after surgery for CT evaluation and were decalcified for HE staining, masson staining and Immunofluorescence staining. **(A)** Micro-CT images for vertebral bodies, distal femurs and Cancellous bone in femurs. **(B–E)** Micro-CT analyses of the bone volume fraction (BV/TV), trabecular number (Tb.N), trabecular thickness (Tb.Th), and trabecular separation (Tb.Sp) for vertebral bodies and distal femurs. **(F)** HE staining and Masson staining for distal femurs. Scale bar = 500 μm. **(G)** Immunofluorescence staining of CD31 and EMCN for distal femurs. Scale bar = 200 μm. All the data were confirmed by three repeated tests. Data were mean ± S.D. *p < 0.05 vs. the aimed group. Sham means that surgery was performed without removing the ovaries and inject intraperitoneally with PBS; Vehicle means that OVX mice were inject intraperitoneally with PBS; MMP2-I1 means that OVX mice were inject intraperitoneally with MMP2-I1 (2 mg/kg, twice per week for 6 weeks).

To assess the effects of MMP2-I1 on osteoporosis, we established an OVX-induced osteoporosis model. The mice were treated with MMP2-I1 for 6 weeks following OVX surgery, and CT was used to assess bone formation (Figure 9A). We found that bone loss is apparent in the vehicle group, whereas MMP2-I1 treatment significantly reversed this trend. In the OVX group, BV/TV, Tb.N, and Tb.Th were decreased in the distal femur and vertebral bodies when compared with those determined in the sham group, whereas Tb.Sp was increased (Figures 9B–E). By contrast, the MMP2-I1 group displayed an increase in BV/TV, Tb.N, and Tb.Th, compared with the vehicle group, whereas the values of Tb.Sp showed the opposite trend (Figures 9B–E). Obvious bone loss was found in H&E- and Masson-stained sections obtained from the OVX groups when compared with those determined in the sham group (Figure 9F). However, the MMP2-I1 group showed reduced OVX-induced bone loss (Figure 9F). Furthermore, immunofluorescence analysis showed that higher expression of CD31 and EMCN was observed in the MMP2-I1 group than in the vehicle group (Figure 9G). These results confirmed that MMP2-I1 treatment attenuated OVX-induced bone loss.

## Discussion

As we know, MMP-2 and MMP-9 are the preferential targets of MMP2-I1. We detected the activity of MMP-2 and MMP-9 in cell lysates and culture media, respectively, under the treatment of MMP2-I1, and found that MMP-2 was the favorite target of MMP2-I1 (Supplementary Figure 3). In this study, we found that MMP2-I1 had positive roles in the osteogenic differentiation of hBMSCs via the p38/MAPK signaling pathway, and in the angiogenesis of HUVECs via the HIF-1α signaling pathway. Use of MMP2-I1 enhanced bone formation and prevented bone loss in the rat tibial defect model and the OVX-induced mouse model of osteoporosis, respectively.

In recent years, many studies have focused on the coupling of angiogenesis and osteogenesis in bone formation, because communication between osteoblasts and endothelial cells is essential for bone healing and remodeling processes (Kusumbe et al., 2014; Xie et al., 2014; Yang et al., 2017; Lin et al., 2018). In the mammalian skeleton, growth of the vascular network is regulated by signals provided by bone cells (Maes et al., 2010). Conversely, the vascular network could influence the osteogenic generation of new bones. For example, various cell types involved in bone metabolism, such as osteocytes, osteoblasts, and osteoclasts can produce or secrete VEGF (Kennedy et al., 2014; Hu and Olsen, 2016; Kim et al., 2018), and VEGF functions as a key inducer of bone vascularization and bone formation (Maes et al., 2010). Understanding the mechanisms linking angiogenesis and bone formation will be relevant in efforts to improve fracture formation or prevent bone mass loss. In this study, the role of MMP2-I1 on hBMSCs during osteogenic differentiation was investigated by qPCR and western blotting, showing that MMP2-I1 facilitated osteo-specific genes and proteins. We found that MMP2-I1 increased ALP activity and promoted mineralization. Furthermore, the effect of MMP2-I1 on HUVECs during angiogenesis was also evaluated by qPCR and western blotting, revealing that MMP2-I1 increased angio-specific mRNAs and proteins. The migration, tube formation, and fibrin gel angiogenesis assays further confirmed the regulatory role of MMP2-I1 on the angiogenesis of HUVECs. These results suggested that MMP2-I1 played a dual role in modulating osteogenesis and angiogenesis *in vitro*. Although MMP2-I1 has no significant dose-dependent or time-dependent manners on osteogenic differentiation and angiogenesis, combined with all our experimental data, we can still see the trends that MMP2-I1 promotes osteogenic differentiation and angiogenesis, hence we reach the above conclusion. In our *in vitro* study, osteogenic differentiation and angiogenesis are two clear research lines, which makes the interaction between them lack sufficient evidence. In the future studies, we should pay attention to the effect of MMP2-I1 on angiogenesis in the process of osteogenesis promotion, as well as the effect of MMP2-I1 on osteogenesis in the process of angiogenesis promotion. These contents can be achieved through relevant experiments such as conditioned medium, which will make our research more convincing.

A recent study showed that a specific endothelium, identified in murine skeletal systems, strongly positive for CD31 and endomucin (CD31^hi^Emcn^hi^), couple angiogenesis and osteogenesis (Kusumbe et al., 2014). This new capillary type is also named type H vessels. As a cell adhesion molecule with proangiogenic activity, PECAM-1/CD31 has been the subject of numerous studies (Park et al., 2015). Endomucin, an endothelial sialomucin closely related to CD34, marks haematopoietic stem cells throughout development (Matsubara et al., 2005). Type H vessels play an important role in the growth of the bone vasculature (Xie et al., 2014). In the present study, we also detected type H vessels (CD31hiEmcnhi) in the OVX-induced mouse model of osteoporosis. We found that more type H vessels were found in the MMP2-I1 treatment group than in the vehicle group. Together with the results of the tibial defect model, we postulate that MMP2-I1 accelerates bone formation via enhancing osteogenesis during coupling with angiogenesis.

Romanchikova et al. (2014) revealed that the MMP-2 inhibitor triazolylmethyl aziridine could reduce melanoma cell invasion and angiogenesis via ERK1/2 phosphorylation. Chemokine (C-C motif) ligand 3 enhances the migration of human chondrosarcoma cells by increasing MMP-2 expression (Hsu et al., 2013). The activation of transforming growth factor (TGF)-β1 by interleukin-13 was blocked using an MMP-2 inhibitor (Firszt et al., 2014). MMP-2 inhibitor III suppressed the invasion of U251 cells in human glioma (Kamino et al., 2011). Moreover, inhibition of MMP-2 abrogated glioma cell migration stimulated by TGF-β2 (Baumann et al., 2009). However, we found that MMP-2 inhibitor 1 did not affect the NF-κB, Wnt/β-catenin, or Smad signaling pathways in hBMSCs during osteogenic differentiation, but regulated the osteogenesis of hBMSCs via activation of the p38/MAPK signaling pathway. In the current study, an inhibitor was used to inhibit the phosphorylation of p38. The levels of osteo-specific mRNAs and proteins, ALP staining, and calcium deposits further verified the adjustive role of the p38/MAPK signaling pathway on the osteogenic differentiation of hBMSCs. Kusumbe et al. (2014) reported that HIF-1α plays a key role in the induction of type H endothelial cells. We also confirmed that MMP2-I1 enhanced the angiogenesis of HUVECs via the HIF-1α signaling pathway.

Numerous studies have investigated the relationship between osteogenesis and angiogenesis in bone healing. However, this is the first study to explore the impact of MMP2-I1 on the osteogenesis of hBMSCs and angiogenesis of HUVECs. However, we did not investigate the role of HIF-1α in the angiogenesis of HUVECs and its function with CD31 and endomucin. Finally, the crosstalk between osteogenesis and angiogenesis was not fully clarified, so further studies are needed.

Our study provides new insight into the positive role of MMP2-I1 during osteogenesis of hBMSCs via the p38/MAPK signaling pathway and angiogenesis of HUVECs via the HIF-1α signaling pathway.

## Data Availability Statement

The raw data supporting the conclusions of this article will be made available by the authors, without undue reservation.

## Ethics Statement

The studies involving human participants were reviewed and approved by Ethics Committee of the Second Affiliate Hospital of Zhejiang University. The patients/participants provided their written informed consent to participate in this study. The animal study was reviewed and approved by Animal Care and Use Committee guidelines of Zhejiang Province.

## Author Contributions

ZP and DX designed research. LJ, KS, and CW performed research and wrote the paper. LJ analyzed data. All authors contributed to the article and approved the submitted version.

## Conflict of Interest

The authors declare that the research was conducted in the absence of any commercial or financial relationships that could be construed as a potential conflict of interest.
