# Supplementary material for: The Effect of MMP-2 Inhibitor 1 on Osteogenesis and Angiogenesis During Bone Regeneration
Source: Front Cell Dev Biol. 2021 Jan 22;8:596783. doi: 10.3389/fcell.2020.596783 (PMC7862568; doi:10.3389/fcell.2020.596783)
Supplement: Supplementary file 1 [file Data_Sheet_1.PDF]

Supplementary Material

1 Supplementary Data

Supplementary Material should be uploaded separately on submission. Please include any supplementary data, figures and/or tables. All supplementary files are deposited to FigShare for permanent storage and receive a DOI.

Supplementary material is not typeset so please ensure that all information is clearly presented, the appropriate caption is included in the file and not in the manuscript, and that the style conforms to the rest of the article. To avoid discrepancies between the published article and the supplementary material, please do not add the title, author list, affiliations or correspondence in the supplementary files.

2 Supplementary Figures

For more information on Supplementary Material and for details on the different file types accepted, please see here. Figures, tables, and images will be published under a Creative Commons CC-BY licence and permission must be obtained for use of copyrighted material from other sources (including re-published/adapted/modified/partial figures and images from the internet). It is the responsibility of the authors to acquire the licenses, to follow any citation instructions requested by third-party rights holders, and cover any supplementary charges.

2.1 Supplementary Figures

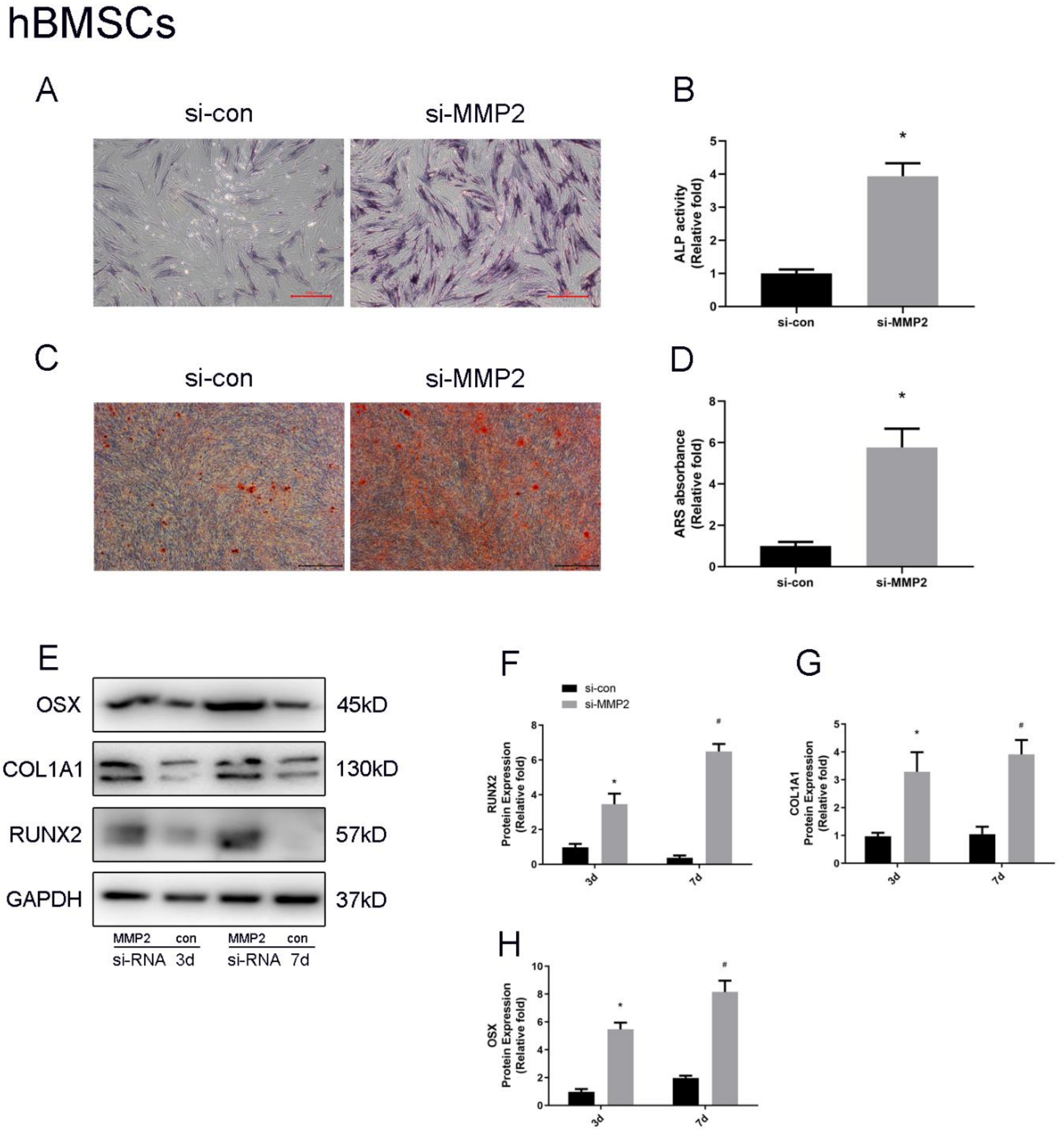

Supplementary Fig. 1 The effects of MMP-2 silence on osteogenesis of hBMSCs
(A) ALP in MMP-2 silenced hBMSCs with MMP-2 siRNA was stained after the osteogenic differentiation for 3 days. (B) The ALP activity of MMP-2 silenced hBMSCs after the osteogenic differentiation for 3 days. (C) Alizarin red staining in MMP-2 silenced hBMSCs after the osteogenic differentiation for 8 days. (D) Mineralization was quantified by the extraction of ARS stain cells. (E-H) The expression of RUNX2, COL1A1 and OSX proteins were determined by Western blot analysis after osteogenic differentiation for 3 and 7 days. All the data were confirmed by three repeated tests. Data were mean ± S.D. * $p < 0.05$ vs. the control group at the same day. # $p < 0.05$ vs. the control group at the same day. Scale bar = 500 μm.

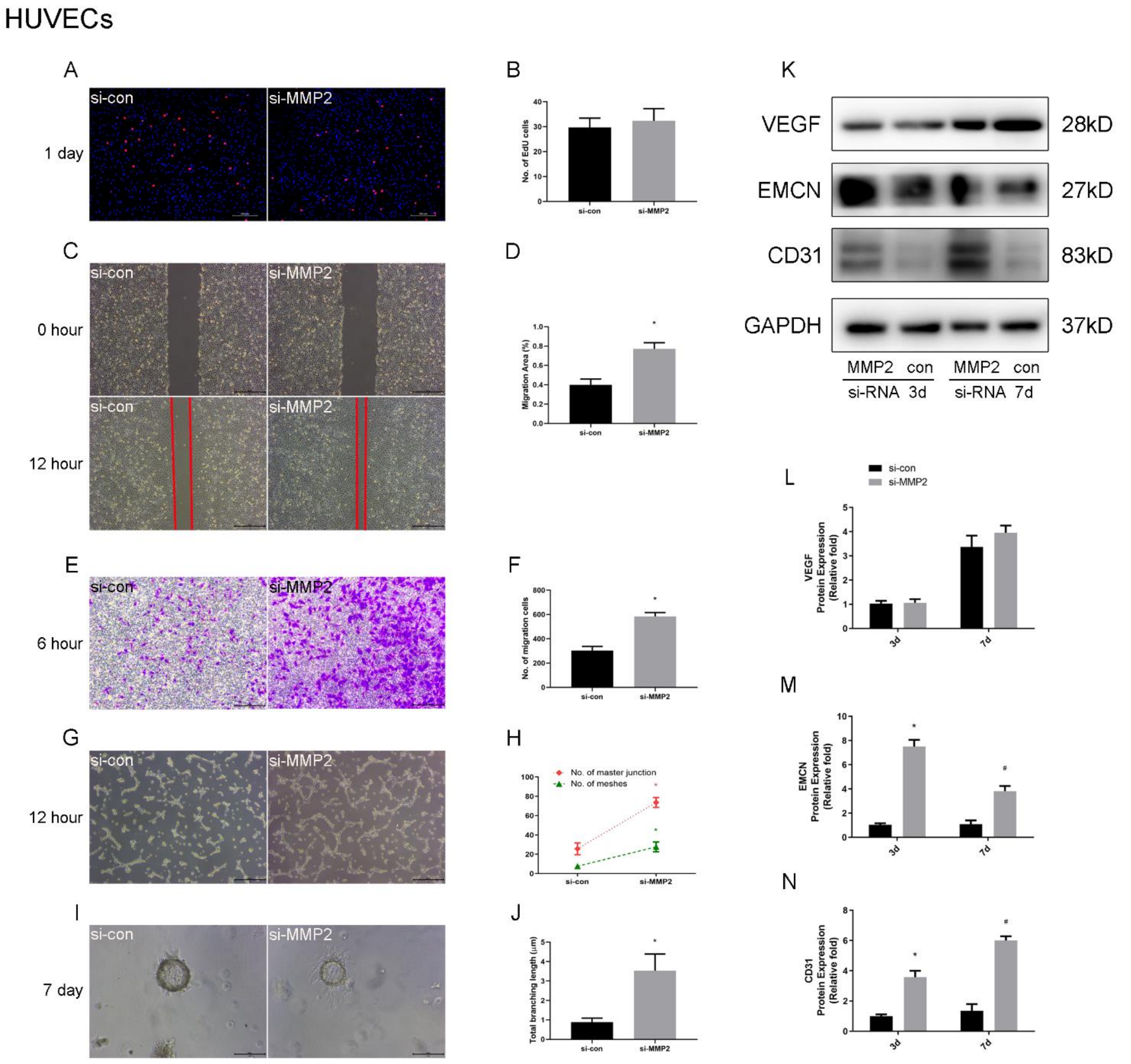

Supplementary Fig. 2 The effects of MMP-2 silence on angiogenesis of HUVECs
(A) EdU-555 fluorescence staining of MMP-2 silenced HUVECs in different groups at day 1. Scale bar = 200 μm. (B) Quantification of EdU-555 positive cells. (C) The scratch wound assay of MMP-2 silenced HUVECs at 0 and 12 h. Scale bar = 500 μm. (D) Quantification of the rate of migration area. (E) The transwell® migration assay of HUVECs at 6 h. Scale bar = 200 μm. (F) Quantification of the migrated cells. (G) The tube formation assay of HUVECs at 12 h. Scale bar = 500 μm. (H) Quantification of meshes and master junction. (I) The fibrin gel angiogenesis assay of HUVECs at 7 d. Scale bar = 200 μm. (J) Quantification of total branching length. (K-N) The expression of VEGF, EMCN and CD31 proteins were determined by Western blot analysis for 3 and 7 days. All the data were confirmed by three repeated tests. Data were mean ± S.D. * $p < 0.05$ vs. the control group at the same day. # $p < 0.05$ vs. the control group at the same day.

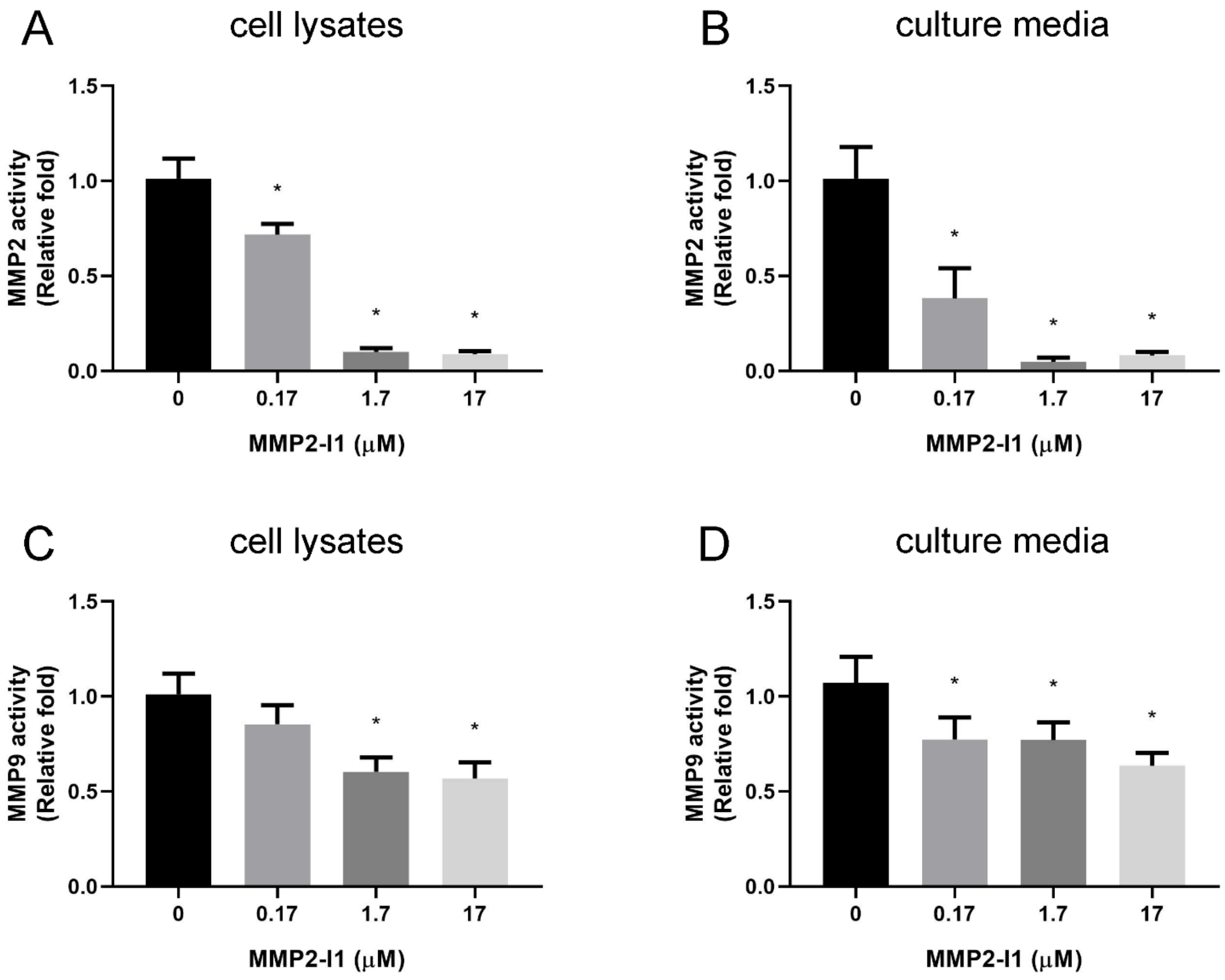

Supplementary Fig. 3 MMP-2 was the favorite target of MMP2-I1
(A) The activity of MMP-2 in hBMSCs under the MMP2-I1 treatment for 3 days. (B) The activity of MMP-2 in the culture media of hBMSCs under the MMP2-I1 treatment for 3 days. (C) The activity of MMP-9 in hBMSCs under the MMP2-I1 treatment for 3 days. (D) The activity of MMP-9 in the culture media of hBMSCs under the MMP2-I1 treatment for 3 days. All the data were confirmed by three repeated tests. Data were mean ± S.D. * $p < 0.05$ vs. the control group.
